# Single-cell delineation of lineage and genetic identity in the mouse brain

**DOI:** 10.1038/s41586-021-04237-0

**Published:** 2021-12-15

**Authors:** Rachel C. Bandler, Ilaria Vitali, Ryan N. Delgado, May C. Ho, Elena Dvoretskova, Josue S. Ibarra Molinas, Paul W. Frazel, Maesoumeh Mohammadkhani, Robert Machold, Sophia Maedler, Shane A. Liddelow, Tomasz J. Nowakowski, Gord Fishell, Christian Mayer

**Affiliations:** 1 Max Planck Institute of Neurobiology, Martinsried, Germany; 2 NYU Neuroscience Institute, Langone Medical Center, New York, NY USA; 3 Broad Institute, Stanley Center for Psychiatric Research, Cambridge, MA USA; 4 Department of Anatomy, University of California, San Francisco, CA USA; 5 Department of Psychiatry, University of California, San Francisco, CA USA; 6 Eli and Edythe Broad Center for Regeneration Medicine and Stem Cell Research, University of California, San Francisco, CA USA; 7 Max Planck Institute of Biochemistry, Martinsried, Germany; 8 Department of Neuroscience and Physiology, New York University Grossman School of Medicine, New York, NY USA; 9 Department of Ophthalmology, New York University Grossman School of Medicine, New York, NY USA; 10 Harvard Medical School, Department of Neurobiology, Boston, MA USA

**Keywords:** Cell fate and cell lineage, Cell type diversity

## Abstract

During neurogenesis, mitotic progenitor cells lining the ventricles of the embryonic mouse brain undergo their final rounds of cell division, giving rise to a wide spectrum of postmitotic neurons and glia^1,2^. The link between developmental lineage and cell-type diversity remains an open question. Here we used massively parallel tagging of progenitors to track clonal relationships and transcriptomic signatures during mouse forebrain development. We quantified clonal divergence and convergence across all major cell classes postnatally, and found diverse types of GABAergic neuron that share a common lineage. Divergence of GABAergic clones occurred during embryogenesis upon cell-cycle exit, suggesting that differentiation into subtypes is initiated as a lineage-dependent process at the progenitor cell level.

## Abstract (web summary)

Single-cell RNA sequencing with parallel tagging of progenitor cells is used to track clonal relationships and transcriptomic signatures during development of the mouse forebrain.

## Main

The central nervous system consists of diverse types of neurons and glia that vary widely in morphology, physiology, connectivity and molecular markers^3,4^. During development, molecular diversity is initially reflected in the regional expression of a narrow set of transcription factors in mitotic progenitors^3^. Transcriptional signatures that distinguish mature neuronal subtypes emerge only after cell-cycle exit and become more sharply defined during postnatal development^1,2,5–7^. The extent to which developmental trajectories are predetermined by specified progenitor lineages during mitotic stages, or emerge through interactions with the environment later in development, remains an open question.

Although previous lineage analyses have elucidated the spatial distribution of clones, they provided little information regarding subtype identities of sister cells^8–12^. More recently, breakthroughs in cellular barcoding strategies and single-cell sequencing^13–21^ have facilitated the recording of lineage tags and gene expression profiles in in vitro systems^22^, in zebrafish^20,23,24^ and in mouse embryogenesis^25^, but have not yet been used to study neurogenesis in the mouse forebrain.

Here we combined high-throughput single-cell RNA sequencing (scRNA-seq) with massively parallel tagging of progenitors to reconstruct lineage relationships during neurogenesis of the forebrain. We focus our analysis on GABAergic neurons, which displayed a surprising degree of clonal divergence among different types of inhibitory neurons. We found that immediately after cell-cycle exit, GABAergic neurons that originated from the same mitotic progenitor diverged into different developmental trajectories. Our findings thus revealed that differentiation into GABAergic subtypes is initiated as a lineage-dependent process at the progenitor cell level.

## Capture of gene expression and lineages

**Fig. 1:**
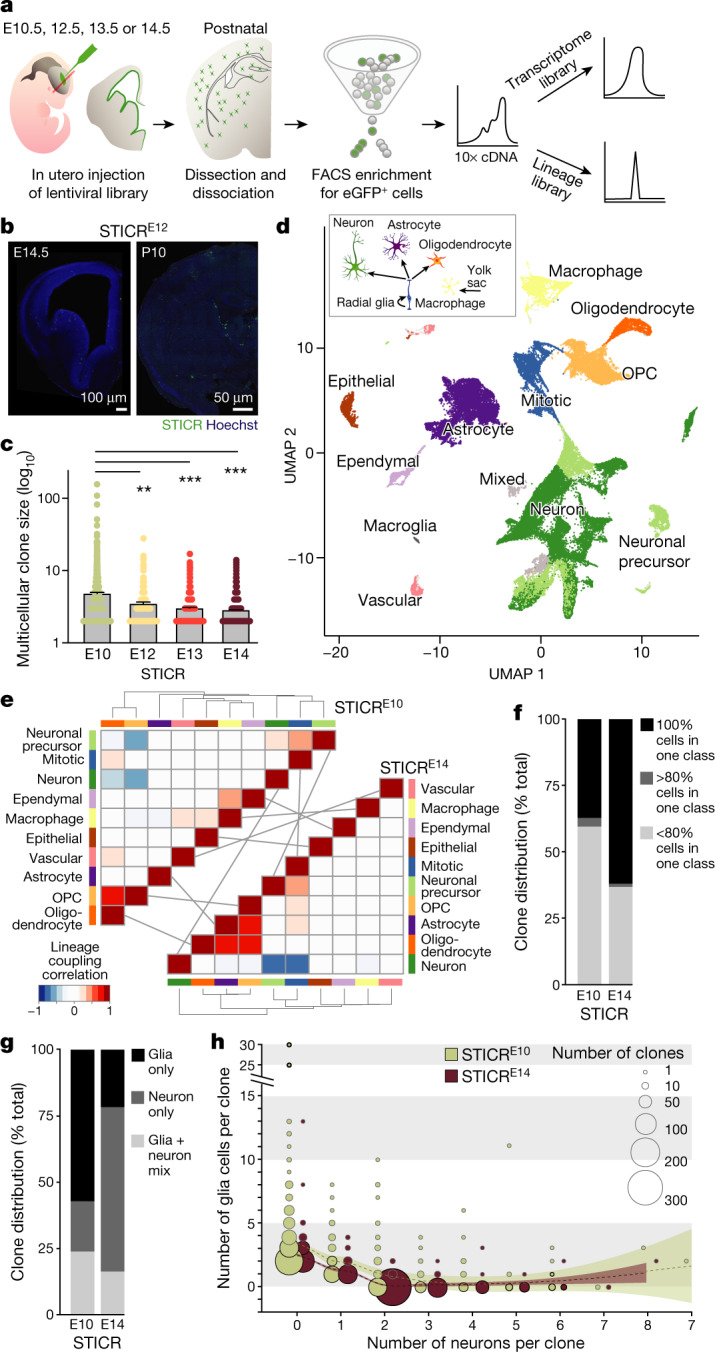
Clonal relationships of forebrain cell classes determined via simultaneous capture of transcriptome and lineage barcodes from single cells. **a**, Schematic of STICR experimental workflow. **b**, Images of coronal brain sections injected with STICR. **c**, Quantification of the average clone size. The error bars indicate s.e.m. Each dot represents a clone. *n* = 1,117 for STICR^E10^, *n* = 169 for STICR^E12^, *n* = 349 for STICR^E13^ and *n* = 1,407 for STICR^E14^. One-way analysis of variance (ANOVA) with Tukey post-hoc test was performed; **P < 0.0015, ***P < 0.0001. **d**, Uniform manifold approximation and projection (UMAP) plot of single cells coloured by cell classes. In the inset, a schematic of cell class origins. **e**, Heatmaps of STICR^E10^ and STICR^E14^ lineage coupling scores between pairs of cell classes, clustered by correlation distance and linkage. The light grey lines link classes across stages. **f**, Quantification of clones with 100%, more than 80%, and less than 80% of cells within a single class. **g**, Quantification of clones containing neurons, glia or a mix. **h**, Number of neurons and glial cells per clone is plotted against one another. The dotted line represents a smooth local regression; the shadow represents the 95% confidence interval.

**Extended Data Fig. 1:**
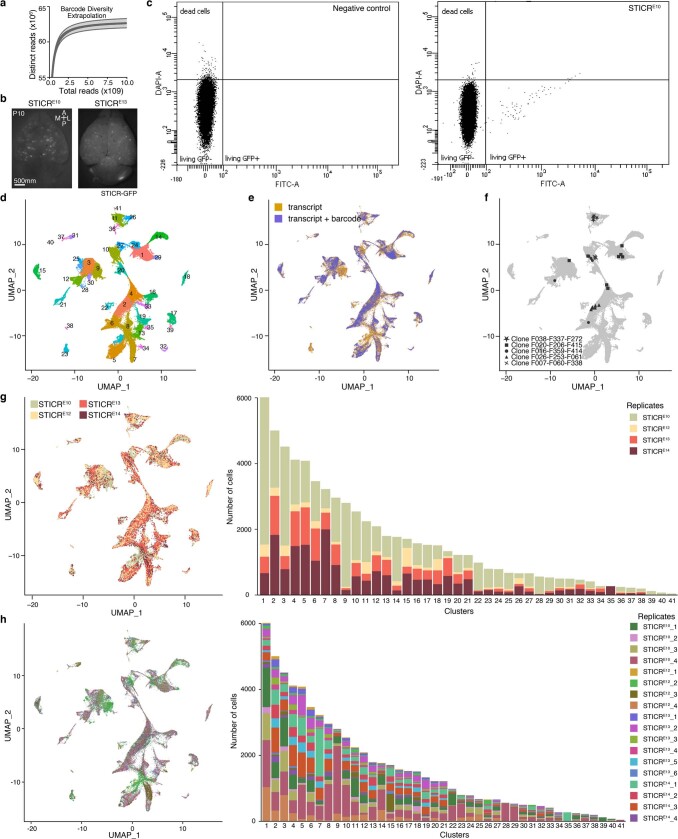
Characterization of STICR datasets. **a**, Barcode diversity extrapolations derived from sequencing ca. 30 million reads of a representative STICR plasmid library. Mean ± 95% CI. **b**, Images of whole mount brains injected with STICR. **c**, Representative FACS gating for negative control (matching non-injected brain) and STICR^E10^. **d**, UMAP plot coloured and numbered for each unsupervised cluster. Each dot represents a single cell. **e**, UMAP plot coloured by cells with recovered transcriptome and cells with recovered transcriptome and lineage barcode. **f**, Examples of clones on UMAP plot. Sibling cell relationships represented by shapes (dot, square, star, cross, triangle). **g**, UMAP plot and bar graph showing the distribution of recovered cells, coloured by stage of injection. **h**, UMAP plot and bar graph showing the distribution of recovered cells, coloured by experimental replicate.

To determine lineage relationships of diverse cell types in the mouse forebrain, we first implemented a lentiviral lineage barcoding method called STICR (scRNA-seq-compatible tracer for identifying clonal relationships; Fig. 1a, Extended Data Fig. 1a, see companion paper^26^), which enables massively parallel tagging of single cells using a high-diversity lentiviral library that encodes synthetic oligonucleotide sequences (lineage barcodes). The STICR tag library was introduced via in utero injections into the lateral ventricles of mouse embryos at embryonic day 10.5 (E10.5; STICR^E10^), E12.5 (STICR^E12^), E13.5 (STICR^E13^) and E14.5 (STICR^E14^), stages that encompass the peak of neurogenesis. This resulted in labelling of mitotic progenitors along the ventricles and their daughter cells that migrated throughout the forebrain, including the cortex, basal ganglia, hippocampus and olfactory bulb (OB) (Fig. 1b, Extended Data Fig. 1b). We waited until postnatal stages when labelled cells differentiated into mature cell types, then dissociated forebrain tissue, FACS-enriched the virally infected cells by selecting for enhanced GFP (eGFP) expression, and performed scRNA-seq with the 10x Chromium System (Fig. 1a, Extended Data Fig. 1c). We analysed transcriptomes from 65,700 high-quality cells that passed filtering (see Methods). To group cells on the basis of patterns of gene expression, we performed a principal components analysis^27^ and batch normalized the different replicates using Harmony^28^, followed by a UMAP visualization and clustering analysis (Extended Data Fig. 1d, Supplementary Data 1), and tracked the position of clonally related cells in the transcriptomic cell-state landscape (Extended Data Fig. 1e–h). The average and maximum size of multicellular clones was larger when the lentiviral library was introduced at E10.5 than at E12.5 or E14.5, when mitotic progenitors presumably undergo fewer divisions (Fig. 1c).

**Extended Data Fig. 2:**
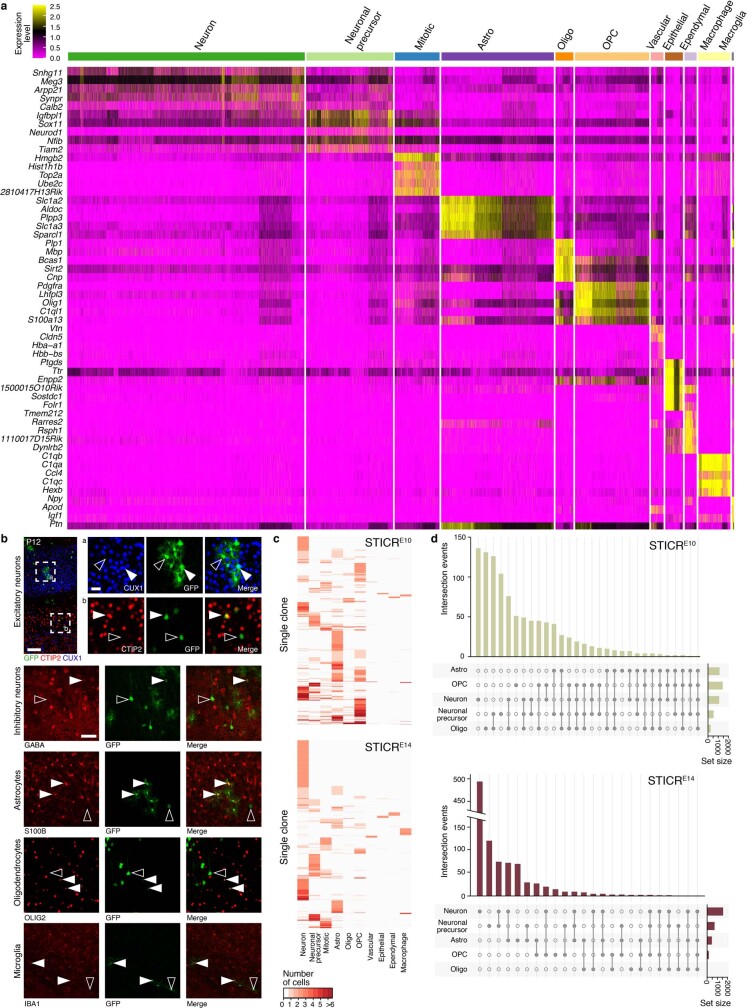
Clonal relationships among cell classes. **a**, Single-cell heatmap showing the normalised expression of the top-5 marker genes for cell classes. Bars are colour-coded based on key in Fig. 1d. **b**, Immunohistochemestry showing STICR^E10^ viral labelling of excitatory (CTIP2/CUX1) and inhibitory (GABA) neurons, astrocytes (S100B), oligodendrocytes (Olig2) and microglia (Iba1). White arrowheads indicate co-labelled cells, empty arrowheads indicate negative cells. Scale bar: 100 μm (top), 30 μm (insets), 50 μm (bottom). Biological replicates, n = 2. **c**, Heatmap showing clonal distributions across cell classes for STICR^E10^ (left) and STICR^E14^ (right). Every horizontal line in the plot is a clone, with number of cells per clone indicated by colour. **d**, UpSet plots displaying clonal intersections between Astrocytes, Neurons, Oligodendrocyte Precursor Cells (OPCs), Neuronal precursors and Oligodentrocytes in STICR^E10^ (top) and STICR^E14^ (bottom). Bar graphs on top indicate the number of observed intersections. The bar graphs on the right depicts total cell number per cell class.

During embryogenesis, asymmetrically dividing radial glia and transiently amplifying mitotic progenitors along the ventricular surface give rise to postmitotic neurons, astrocytes and oligodendrocytes^3,4,29–32^ (Fig. 1d). To quantify clonal relationships between cell classes, we assigned the 41 clusters into cell classes (Fig. 1d) based on the co-expression of multiple marker genes (Extended Data Fig. 2a, b, Supplementary Data 2) and counted the distribution of STICR tags across cell classes (Extended Data Fig. 2c, d). Next, we assessed the likelihood of recovering shared lineage barcodes from all pairs of cell classes and quantified lineage coupling by calculating a z-score for clone counts with respect to a random distribution (see Methods)^24^. Hierarchical clustering of the pairwise correlation between coupling scores revealed structured groups, which comprised clonally related cell classes (Fig. 1e). STICR^E10^ and STICR^E14^ clones stayed predominantly within a class (Fig. 1e). However, STICR^E10^ showed stronger coupling between oligodendrocytes and oligodendrocyte precursor cells (OPCs), whereas STICR^E14^ showed stronger coupling between oligodendrocytes, OPCs and astrocytes (Fig. 1e). Notably, 37.2% of STICR^E10^ clones contained cells of a single class, compared with 62% of STICR^E14^-descent clones (Fig. 1f). Moreover, 57.2% of STICR^E10^ clones were glial clones, 19% were neuronal clones and 23.8% were mixed clones (that is, clones with cells spanning multiple cell classes) versus 21.7%, 62% and 16.3%, respectively, for STICR^E14^ (Fig. 1g). STICR^E10^ mitotic progenitors produced larger clones, with up to 30 sister cells per clone, compared to STICR^E14^ mitotic progenitors, which produced up to 13 sister cells (Fig. 1h). Together, early-labelled progenitors generated a higher proportion of clones that dispersed across multiple cell classes than late-labelled progenitors that produced a majority of neuronal clones, consistent with progressive temporal fate specification of progenitors.

## Clonal convergence and divergence

**Fig. 2:**
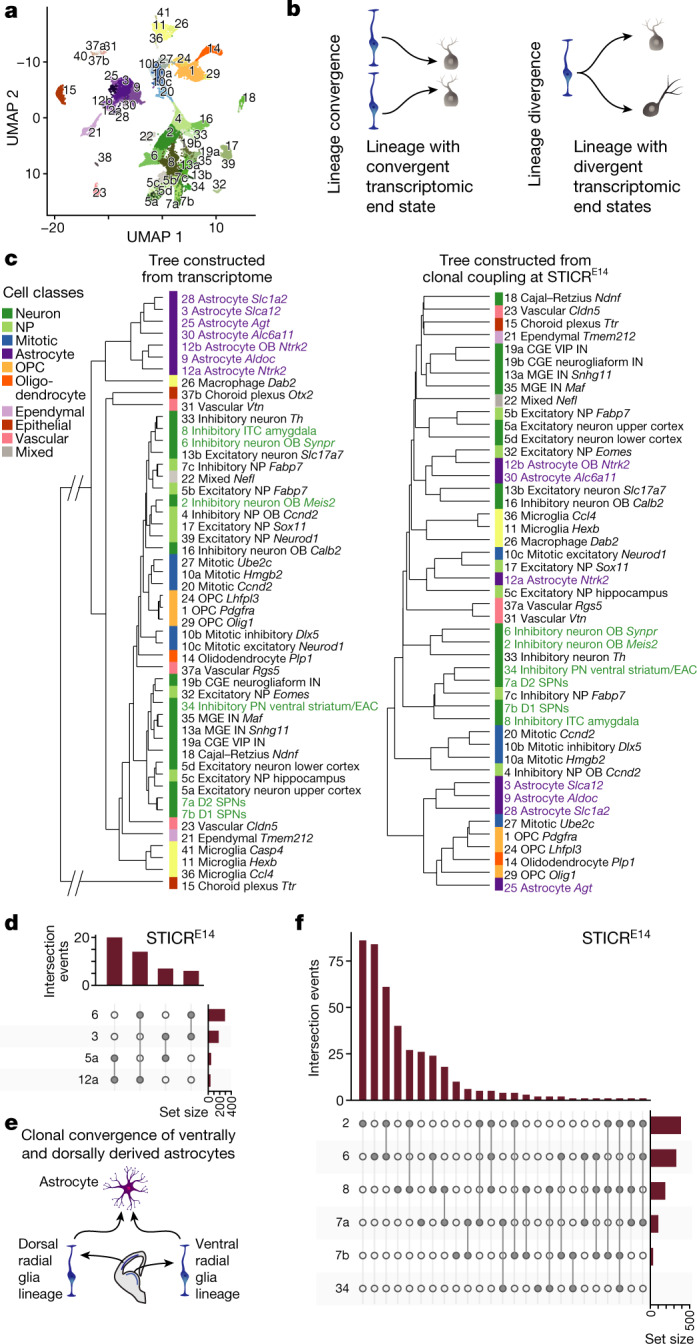
Lineage convergence and divergence in the mouse forebrain. **a**, UMAP plot of single cells from the forebrain coloured by cluster. **b**, Schematics of patterns of lineage convergence and divergence. **c**, Dendrograms representing cluster relationships based on transcriptomic similarity (left) and lineage coupling correlations (STICR^E14^, right). In purple are examples of lineage convergence; in green are examples of lineage divergence. CGE, caudal ganglionic eminence; EAC, central extended amygdala; IN, interneuron; MGE, medial ganglionic eminence; NP, neuronal precursor; PN, projection neuron; VIP, vasoactive intestinal polypeptide. **d**, UpSet plot of selected intersections for STICR^E14^. Only dispersing clones are shown. The bar graph at the top shows the number of observed intersections, and the bar graph on the right shows the number of cells per cluster. **e**, Schematic for lineage convergence of astrocyte clusters 3 and 12a. **f**, UpSet plot for selected ventral inhibitory neuron types in STICR^E14^. The bar graph at the top shows the number of observed intersections, and the bar graph on the right shows the number of cells per cluster.

**Extended Data Fig. 3:**
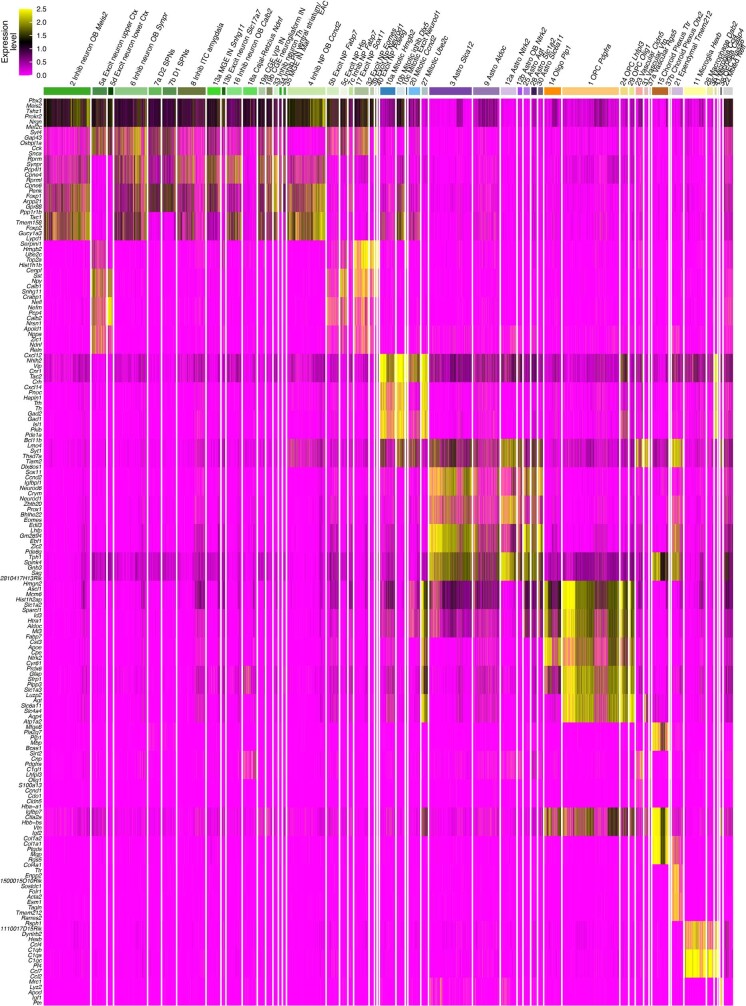
Differential gene expression analysis of cell type clusters. Single-cell heatmap showing the normalised expression of the top-5 marker genes for cell types. Bars are colour-coded based on key in Fig. 2a. Astro, astrocyte; CGE, caudal ganglionic eminence; Ctx, neocortex; EAC, central extended amygdala; Excit, excitatory; Hip, hippocampus; IN, interneuron; Inhib, inhibitory; MGE, medial ganglionic eminence; NP, neuronal precursor; OB, olfactory bulb; Oligo, oligodendrocyte; OPC, oligodendrocyte precursor cell; PN, projection neuron; SPN, spiny projection neuron.

**Extended Data Fig. 4:**
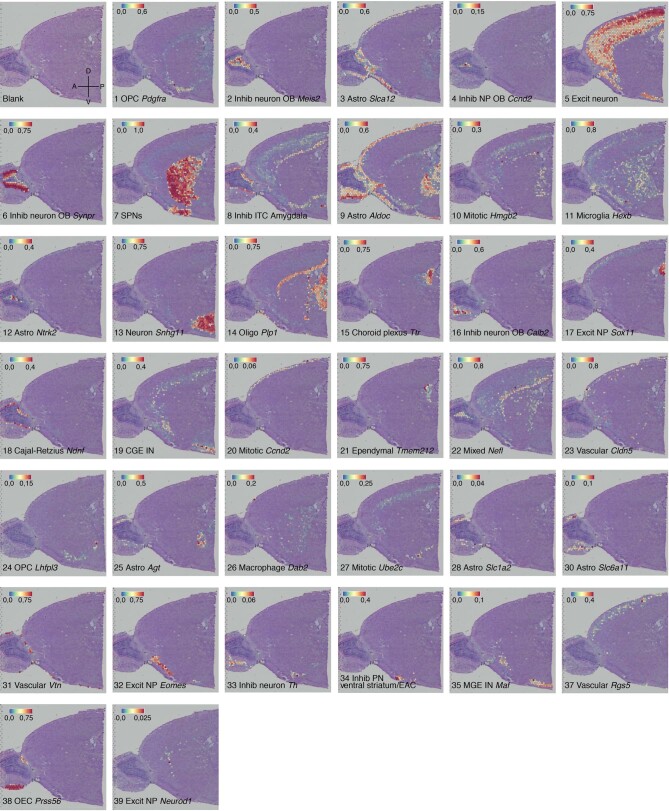
Spatial transcriptomic mapping of cell type clusters. Spatial transcriptomic mapping showing localization of cell type clusters. Astro, astrocyte; CGE, caudal ganglionic eminence; Ctx, neocortex; EAC, central extended amygdala; Excit, excitatory; Hip, hippocampus; IN, interneuron; Inhib, inhibitory; MGE, medial ganglionic eminence; NP, neuronal precursor; OB, olfactory bulb; Oligo, oligodendrocyte; OPC, oligodendrocyte precursor cell; PN, projection neuron; SPN, spiny projection neuron.

**Extended Data Fig. 5:**
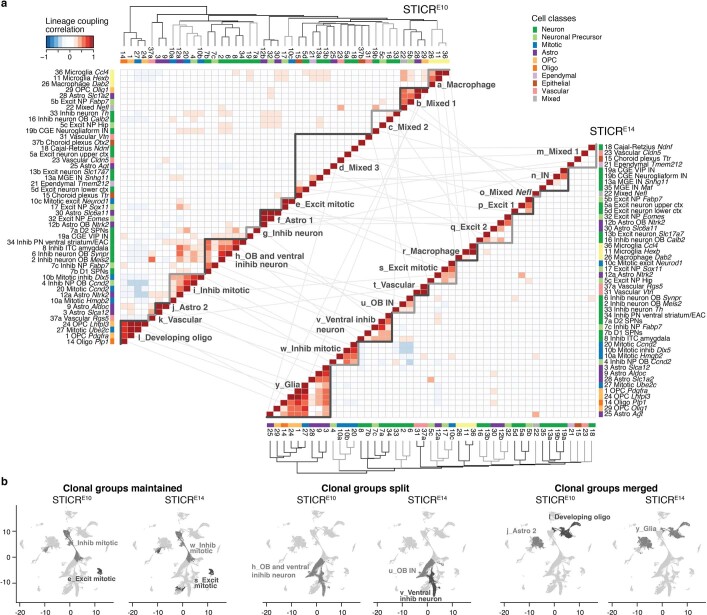
Clonal coupling of forebrain cell types. **a**, Heatmaps of STICR^E10^ (left) and STICR^E14^ (right) lineage coupling scores between pairs of cell types, clustered by correlation distance and linkage. Values range from positive (red, coupled) to negative (blue, anti-coupled). Light-grey lines link clusters across stages. Clonal groups are surrounded with grey lines and manually annotated (a-y). Astro, astrocyte; CGE, caudal ganglionic eminence; Ctx, neocortex; EAC, central extended amygdala; Excit, excitatory; Hip, hippocampus; IN, interneuron; Inhib, inhibitory; MGE, medial ganglionic eminence; NP, neuronal precursor; OB, olfactory bulb; Oligo, oligodendrocyte; OPC, oligodendrocyte precursor cell; PN, projection neuron; SPN, spiny projection neuron. **b**, Feature plots highlighting examples of clonal groups that are maintained (left), split (middle) or merged (right) on the UMAP plot for STICR^E10^ and STICR^E14^.

Our analysis thus far focused on the lineage relationships among cell classes. To gain higher resolution, we next explored clonal relationships between finer-grained subtypes. The 41 clusters were annotated on the basis of marker gene expression and mapped to anatomical brain regions using Visium Spatial Gene Expression^33^ (Extended Data Figs. 3, 4, Supplementary Data 3). Of these, nine clusters were reclustered to gain a higher level of detail (for example, cluster 7 was split into clusters 7a and 7b; Fig. 2a). Hierarchical clustering of the pairwise correlation between coupling z-scores revealed structured groups of clusters (‘clonal groups’ a–y; Extended Data Fig. 5).

**Extended Data Fig. 6:**
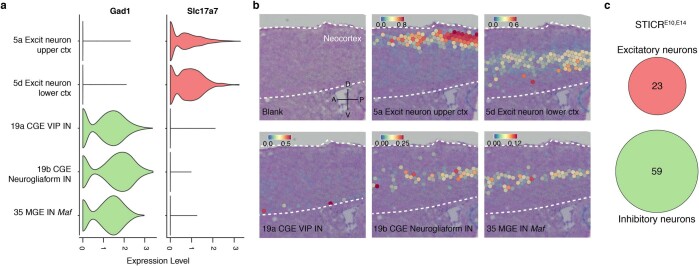
Excitatory and inhibitory lineages do not overlap in the murine cortex. **a**, Violin plots showing the normalised marker gene expression Slc17a7 (marker gene for excitatory neurons) and Gad1 (marker gene for inhibitory neurons). CGE, caudal ganglionic eminence; Ctx, neocortex; Excit, excitatory; IN, interneuron; Inhib, inhibitory; MGE, medial ganglionic eminence. **b**, Spatial transcriptomic mapping showing localization of cortical neuron clusters in the mouse brain. **c**, Venn diagram quantifying the number of clones within excitatory and inhibitory clusters.

First, we tested whether excitatory and inhibitory neurons in the neocortex originate from the same progenitor. We identified clusters of cortical excitatory and inhibitory neurons based on the expression of canonical marker genes (*Gad1* inhibitory and *Slc17a7* excitatory) and Visium Spatial Gene Expression, and quantified their clonal relationship (Extended Data Fig. 6). In contrast to Delgado et al.^26^ in the same issue, who found clonally related GABAergic and glutamatergic neurons in humans, we observed no evidence for shared lineages of excitatory and inhibitory cortical neurons in STICR^E10^ or STICR^E14^ mice (Extended Data Fig. 6c).

**Extended Data Fig. 7:**
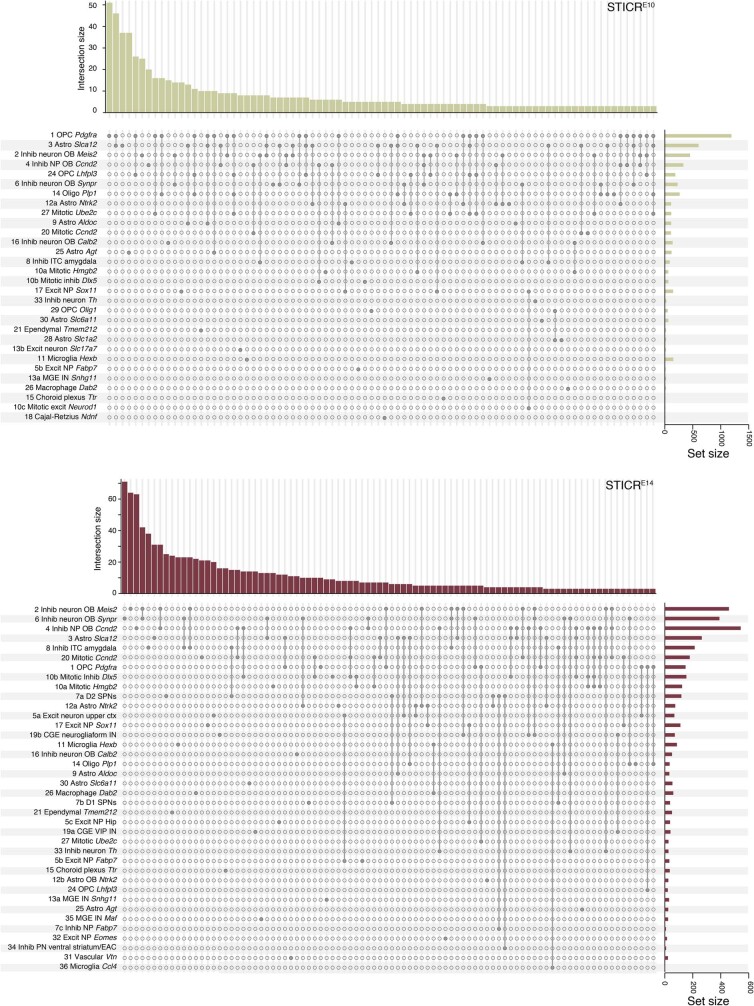
Clonal dispersion across cell types. UpSet plots displaying intersections for cell type clusters in STICR^E10^ (top) and STICR^E14^ (bottom). Bar graphs on top indicate the number of observed intersections. The total cell number per cluster is shown on the right bar graphs. Intersection cut-off was set to 3 to fit on the plot. Astro, astrocyte; CGE, caudal ganglionic eminence; Ctx, neocortex; EAC, central extended amygdala; Excit, excitatory; Hip, hippocampus; IN, interneuron; Inhib, inhibitory; MGE, medial ganglionic eminence; NP, neuronal precursor; OB, olfactory bulb; Oligo, oligodendrocyte; OPC, oligodendrocyte precursor cell; PN, projection neuron; SPN, spiny projection neuron.

Next, we tested whether developmental histories can be predicted from the assumption that cell types with transcriptomically similar identities are clonally related. This assumption may not always hold true, because similar cell states could arise from different lineages (that is, convergence) and distinct cell states could share a lineage (that is, divergence) (Fig. 2b). To explore these possibilities, we compared hierarchies constructed from transcriptome information and hierarchies constructed from lineage coupling correlations (Fig. 2c). Seven astrocyte clusters appeared transcriptomically similar to one another, as they occupied a single clade on the transcriptome dendrogram, but largely belonged to different clades on the lineage dendrogram (Fig. 2c, clusters highlighted in purple). Moreover, we found that astrocyte lineages of the dorsal forebrain and astrocyte lineages of the ventral forebrain converged on seemingly identical astrocyte subtypes. In particular, clusters ‘3 Astrocyte *Slca12*’ and ‘12a Astrocyte *Ntrk2*’ were both clonally related to clusters ‘6 Inhibitory neuron OB *Synpr*’ and ‘5a Excitatory neuron upper cortex’ (Fig. 2d, Extended Data Fig. 7), which suggests that distinct ventral and dorsal radial glia can give rise to transcriptomically similar astrocyte populations (Fig. 2e). Another example of convergence was OB neuroblasts (‘4 Inhibitory NP OB *Ccnd2*’) and dentate gyrus neuroblasts (‘17 Excitatory NP *Sox11*’), which occupied a single clade on the transcriptome dendrogram but distant clades on the lineage dendrogram (Fig. 2c). Thus, cells originating from distinct mitotic progenitors located in different brain areas can converge to similar transcriptomic identities.

**Extended Data Fig. 8:**
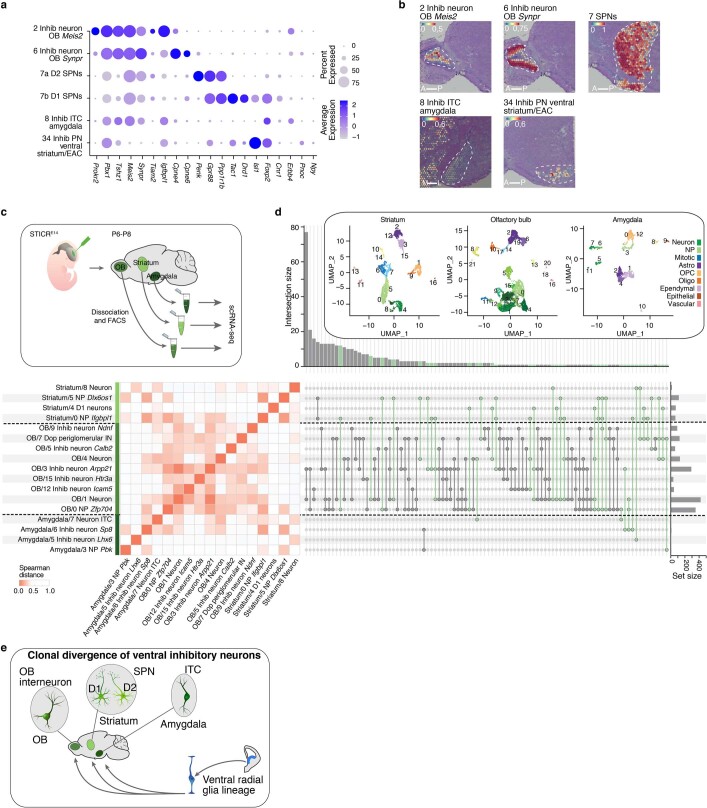
Lineage divergence in ventral inhibitory neurons. **a**, Dot plot showing gene expression markers of ventral inhibitory neuron clusters. The size of the dots indicates the percentage of cells expressing a specific marker gene. The colour of the dots represents the average expression level. EAC, central extended amygdala; Inhib, inhibitory; MGE, medial ganglionic eminence; OB, olfactory bulb; PN, projection neuron; SPN, spiny projection neuron. **b**, Spatial transcriptomic mapping showing localization of ventral inhibitory neuron clusters in the mouse brain. **c**, Schematic representing the workflow for the manual dissection of striatum, olfactory bulb (OB), and amygdala from P6-P8 brains injected with STICR^E14^. **d**, UpSet plot displaying clonal intersections amongst and within neuronal clusters of striatum, OB and amygdala. Only dispersing clones are shown. Bar graph on top indicate the number of observed intersections. The total cell number per cluster is represented on the right bar plot. Intersections amongst striatum, OB and amygdala are coloured in green. The heatmap shows the Spearman distance between the log-normalized average cluster gene expression. The insets (top right) are UMAP plots of single cells from striatum, OB and amygdala datasets, with clusters coloured by cell class type. NP, neuronal precursor. **e**, Schematic of lineage divergence for ventral inhibitory neuron cell types.

The most striking example of clonal divergence was observed for inhibitory neuron clones, which are known to derive from mitotic progenitors in the ganglionic eminences of the ventral forebrain (Fig. 2c, clusters highlighted in green). In particular, we identified six GABAergic projection neuron and interneuron clusters of the subpallium and OB that displayed high lineage coupling (Fig. 2c, Extended Data Fig. 5, see ‘clonal groups’ h,u,v). Neurons within these clusters included direct (D1) and indirect (D2) spiny projection neurons (SPNs) of the striatum (clusters 7b and 7a, respectively), projection neurons of the central extended amygdala (cluster 34), intercalated cells (ITCs) of the amygdala (cluster 8) and OB interneurons (clusters 2 and 6; Extended Data Fig. 8a, b). These GABAergic neurons were clonally related, although they showed drastically different transcriptomic profiles (Fig. 2c, f, Extended Data Figs. 7, 8a) and are known to have different morphologies, connectivity patterns and occupy different brain regions^3^. For example, ITCs of the amygdala were clonally related to interneurons of the OB and multiple GABAergic projection neuron types, including SPNs of the striatum and central extended amygdala. Moreover, both D1 and D2 SPNs were clonally related to interneurons of the OB (Fig. 2f). Next, we created three additional STICR^E12^ datasets of anatomically dissected brain regions (the OB, striatum and amygdala) and measured the similarity between the average gene expression of clusters (Extended Data Fig. 8c, d). Clones of GABAergic neurons were distributed across different forebrain structures and frequently across transcriptomically diverse subtypes. Thus, individual progenitors of GABAergic neurons can give rise to a wide range of different GABAergic subtypes (Extended Data Fig. 8e).

## Embryonic divergence of GABAergic neurons

**Fig. 3:**
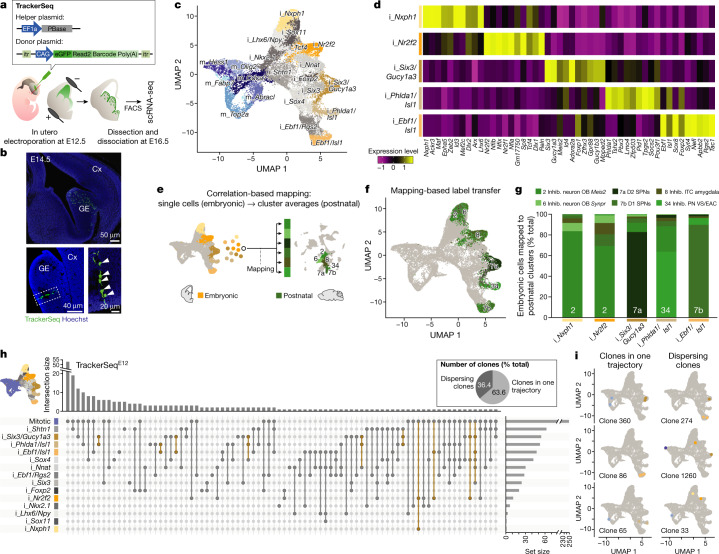
Newly born GABAergic sister cells diverge into different precursor states. **a**, Schematic of the TrackerSeq experimental workflow. PBase, *piggyBac* transposase. **b**, Images of coronal brain sections electroporated with TrackerSeq^E12^ and collected at E14.5. Cx, cortex; GE, ganglionic eminence. Magnification on the bottom right panel shows a radial cluster of newborn cells (white arrowheads). **c**, UMAP plot of integrated embryonic scRNA-seq datasets, coloured by clusters. i, inhibitory; m, mitotic. **d**, Heatmap showing the normalized expression of the top ten marker genes for the five precursor states. **e**, Schematic of the strategy for computationally mapping embryonic precursor state cells to postnatal clusters. **f**, UMAP of the embryonic dataset, with precursor state cells coloured based on the mapping results. **g**, Bar graph quantifying the correlation-based mapping of cells from the five precursor states to selected postnatal ventral GABAergic neuron clusters. The numbers on the bars indicate the dominant mapped postnatal cluster. Inhib., inhibitory; VS, ventral striatum. **h**, UpSet plot for all intersections of TrackerSeq^E12^. The bar graph on the top shows the number of intersections. Mitotic clusters were merged in a single cluster (mitotic). The total cell number per cluster is represented in the bar graph on the right. Intersections among precursor states are coloured in ochre. The bars are colour-coded according to Fig. 3c. The inset in the top right represents the percentage of multicellular clones that follow a single trajectory or dispersed across several precursor state trajectories. **i**, Examples of clones where sibling cells traverse a single developmental trajectory (left) or different trajectories (right) on the UMAP.

**Extended Data Fig. 9:**
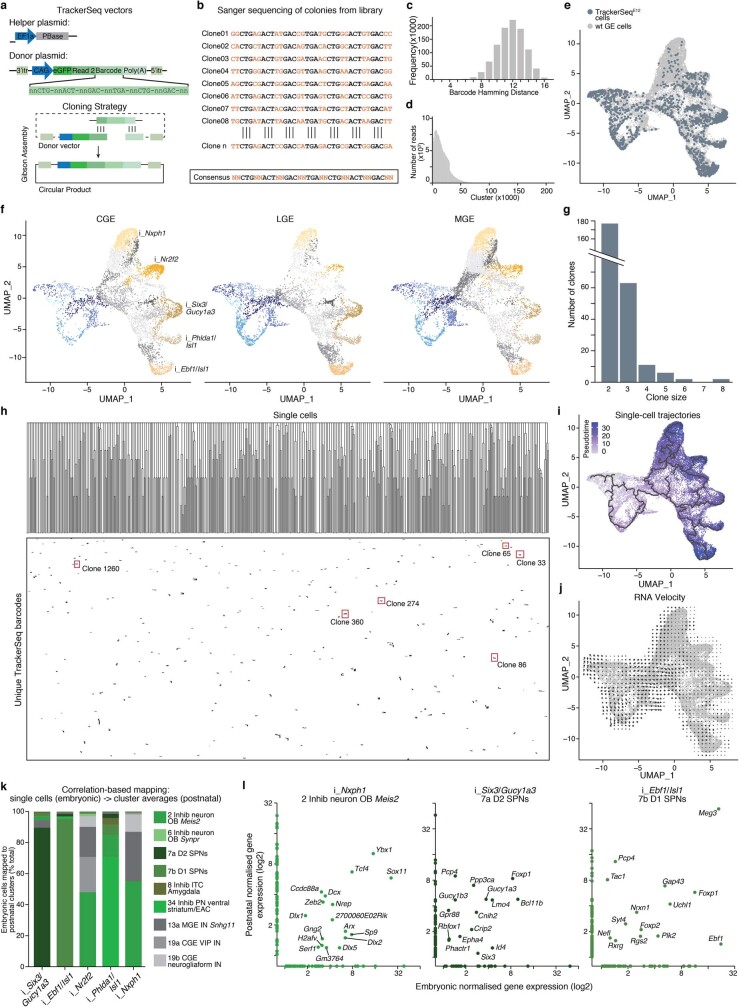
Characterization of TrackerSeq. **a**, Vector maps and cloning strategy of TrackerSeq. PBase: *piggyBac* transposase. **b**, Sanger sequencing results of individual *E. coli* colonies ('clones'), depicting the consensus sequence of the TrackerSeq lineage barcode. **c**, Pairwise hamming distance of 1000 barcodes randomly sampled from the TrackerSeq library. **d**, ~3.6 x 10^6^ raw sequencing reads were collapsed into ~2 x 10^5^ clusters, where each cluster is defined as a unique lineage barcode. **e**, UMAP plot of embryonic scRNA-seq datasets, cells coloured by dataset type (blue, TrackerSeq; grey, wild type). **f**, UMAP plot of single cells from the caudal, lateral and medial ganglionic eminences (CGE, LGE, MGE) of wild type (wt) embryos, coloured by clusters from Fig. 3c. **g**, Histogram showing distribution of clone sizes for TrackerSeq dataset. **h**, Clustered heatmap of TrackerSeq^E12^ barcodes. Rows are single GABAergic precursor cells for which both transcriptome and >1 TrackerSeq barcodes were retrieved; column represents unique TrackerSeq barcodes. Highlighted barcodes are those represented in Fig. 3i. **i**, Developmental trajectories of single-cell transcriptomes, coloured by pseudotime score. **j**, RNA Velocity plot. Arrows direction represent prediction of cells’ future gene expression. **k**, Bar graph quantifying the correlation-based mapping of cells from the 5 embryonic precursor states to clusters of postnatal GABAergic forebrain neurons, including cortical interneuron types. EAC, central extended amygdala; IN, interneuron; Inhib, inhibitory; NP, neuronal precursor; OB, olfactory bulb; PN, projection neuron; SPN, spiny projection neuron. **l**, Scatter plots showing the normalized average cluster gene expression of the top 100 marker genes for a selected embryonic cluster and the top 100 marker genes for a selected postnatal cluster. Clusters were selected based on the mapping efficiency.

To test whether clonal divergence of GABAergic subtypes is the result of early fate specification within embryonic progenitor zones, or rather emerges during postnatal development, we studied single-cell lineage histories when molecular diversity of cell types first occurs. Because STICR labels mitotic progenitors indiscriminately along the embryonic ventricles, it is not suited to deliver a large number of lineage tags to a spatially defined region. To tag mitotic progenitors specifically in the ganglionic eminences, we developed a transposon-based barcoding approach (TrackerSeq; Fig. 3a, Extended Data Fig. 9a–d; see Methods) that uses the *piggyBac* transposon system to randomly integrate an eGFP reporter cassette into the genome of electroporated mitotic progenitors^24,34^. A DNA sequence containing random nucleotides was cloned into the 3′ untranslated region of eGFP, making it detectable by scRNA-seq.

We targeted TrackerSeq to ganglionic eminence progenitors at E12.5, FACS-enriched electroporated cells at E16.5, and performed scRNA-seq (Fig. 3a, b). Hierarchical clustering of TrackerSeq DNA tags organized cells into 256 distinct multicellular clones of GABAergic neurons (Extended Data Fig. 9e–h). To gain a high resolution of embryonic cell states in the ganglionic eminences, we integrated the TrackerSeq datasets with wild-type scRNA-seq datasets that we collected at E13.5 and E15.5 from the medial, caudal and lateral ganglionic eminences (Fig. 3c, Extended Data Fig. 9e, f). We performed a pseudotime trajectory analysis using Monocle3^35^, which is a diffusion pseudotime algorithm that learns the sequence of gene expression changes and identifies developmental branch points (Extended Data Fig. 9i). In addition, we used RNA velocity^36^, a method that compares the ratios of unspliced and spliced mRNA per gene, to predict the direction and speed of cell-state transitions across the trajectories (Extended Data Fig. 9j).

From a common pool of mitotic progenitors, five different trajectories (that is, precursor states) of postmitotic inhibitory neurons emerged soon after cell-cycle exit, which we named after the top marker genes that these clusters expressed (‘i_*Six3*/*Gucy1a3*’, ‘i_*Ebf1*/*Isl1*’, ‘i_*Phlda1*/*Isl1*’, ‘i_*Nr2f2*’ and ‘i_*Nxph1*’; Fig. 3c, d). We used a correlation-based distance metric (see Methods) to map cells from each embryonic precursor state to inhibitory clusters of the postnatal STICR dataset (Fig. 3e–g, Extended Data Fig. 9k). For example, 83% of cells from the trajectory ‘i_*Six3*/*Gucy1a3*’ mapped to the postnatal cluster ‘7a D2 SPNs’, and 89% of cells from trajectory ‘i_*Ebf1*/*Isl1*’ mapped to cluster ‘7b D1 SPNs’ (Fig. 3g), suggesting an early emergence of postnatal signatures. Consistent with this idea, OB interneuron precursors, as well as D1 and D2 striatal precursors, maintained multiple marker genes through development (Extended Data Fig. 9l).

**Extended Data Fig. 10:**
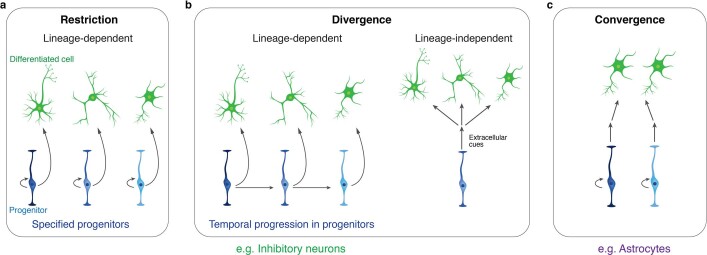
The contribution of lineage to the generation of cell diversity. Schematic illustrating different scenarios of how cellular diversity could arise in the brain. Different cell types could arise from specified or fate-restricted progenitor cells (a), or be born sequentially from a common pool of progenitor cells (b, left). Such lineage-dependent processes suggest that cell-intrinsic mechanisms or local cues at the mitotic progenitor level, determine the fate of newborn cells. As another option, lineage-independent mechanisms such as extracellular induction, activity-dependent processes, or stochastic regulation (b, right) could drive the differentiation into different subtypes. Lineage-dependent and independent mechanisms are not mutually exclusive. c) Convergence is the process by which similar cell states arise from different lineages.

We next asked whether clonally related cells traverse the same or different trajectories. Notably, while cells of 63.6% of clones entered the same trajectory, 36.4% of the clones diverged into differing trajectories shortly after they exited the cell cycle (Fig. 3h, i). For example, we found sister cells located on the ‘7b D1 SPN’ and ‘7a D2 SPN’ trajectories, ‘7a D2 SPN’ and ‘8 Inhibitory ITC–amygdala’ trajectories, and the ‘2 Inhibitory neuron OB *Meis2*’ and ‘8 Inhibitory ITC–amygdala’ trajectories (Fig. 3h, i). Taken together, these data show that progenitor cells in the ganglionic eminences can produce daughter cells that traverse different developmental trajectories during peak neurogenesis. This suggests that clonal divergence into different GABAergic precursor states is initiated at the level of mitotic progenitor cells and thus as a lineage-dependent process (Extended Data Fig. 10).

## Discussion

During neurogenesis, a wide range of postmitotic neurons and glia arise from mitotic progenitors lining the embryonic ventricles. The extent to which developmental histories of mature cell types can be predicted on the basis of the assumption that cell types with transcriptomically similar identities are developmentally related has remained obscure. Using methods that simultaneously capture transcriptomic signatures and lineage histories of individual cells, we found both transcriptomically similar cell types that arose from different lineages (that is, convergence) and transcriptomically distinct cell types that share a clonal relationship (that is, divergence). The most striking example of divergence was GABAergic clones stemming from mitotic progenitors in the ventral forebrain. These clones did not only disperse into distinct brain regions, but also diverged into types with grossly different transcriptomic signatures. While perhaps some degree of clonal divergence would be expected early in neurogenesis at a time when a large number of radial glial progenitors undergo symmetric proliferative divisions, our results reveal clonal divergence at later stages of neurogenesis.

In agreement with Mayer et al.^1^, we found that in all ganglionic eminences, newborn GABAergic neurons diverge into different precursor states after cell-cycle exit. It was not clear whether clonally related sister cells enter the same or different precursor states (Extended Data Fig. 10). Because our new methods capture clonal histories, we were able to address this question and found that newborn sister cells often diverged in different trajectories, suggesting that mechanisms on the level of progenitors delineate genetic identity and ultimately cell fate^37,38^. The ganglionic eminences can be divided into more than a dozen progenitor subdomains that are uniquely demarcated by the combinatorial expression of transcription factors^39,40^. The superimposition of a cellular mechanism that gives rise to different postmitotic signatures with domain-specific factors and morphogens provides a large combinatorial framework that could explain how an enormous diversity of inhibitory types is generated in the forebrain. Whether the sequential production of different types follows a stereotypic sequence, or involves stochastic events that occur during cell-cycle exit, remains unknown. One possibility is that the sequential production of cell types depends on the interaction of progenitors with developmentally dynamic transcription factors and morphogens.

While recent work has examined how cell populations vary across species^41^, the methods developed in our study and Delgado et al. ^26^ in the same issue enable comparison of developmental histories of cell types across mouse and human. The identification of species-specific lineages will provide insight into the evolution of cellular diversity. Both STICR and TrackerSeq capture partial clones, which is sufficient to detect clonal divergence and convergence. However, at present, our methods can infer but not prove lineage restriction.

Somatic mutations, which are clonally inherited, contribute to numerous neurodevelopmental diseases^42–47^. Determining lineage relationships might explain why certain subsets of cells are affected in developmental disorders. Looking forward, we anticipate that studies combining lineage information with genetic mutations will facilitate the explorations of such clinical phenotypes.

## Methods

### STICR lentiviral library preparation and validation

We synthesized a high-complexity lentivirus barcode library that encodes approximately 60–70 million distinct oligonucleotide RNA sequences (STICR barcodes). STICR barcodes comprised three distinct oligonucleotide fragments cloned sequentially into a multicloning site within the 3′ UTR of an enhanced green fluorescent protein (eGFP) transgene under control of a ubiquitous CAG promoter in a modified lentiviral plasmid (pSico, Addgene, #11578). Each barcode fragment was derived from one of three oligonucleotide pools comprising 500 distinct sequences, allowing for up to 125 million unique combinatorial barcode sequences (500^3^). Following the ligation of each oligonucleotide fragment into the multicloning site, the plasmid library was electroporated into electrocompetent MegaX DH10B cells (Fisher, #C640003) and grown for 12 h overnight at 37 °C on LB agar plates (Fisher, BP1425-500) with carbenicillin (Fisher, #BP26481). The resulting colonies were scraped and plasmid extraction was performed using a midi-prep kit (Macherey Nagel, 740412.5). This process was repeated until all three barcode fragments were added.

Lentivirus production was performed by first transfecting HEK293 cells with the barcode library along with lentiviral helper plasmids pMDLg/pRRE (Addgene, #12253), pRSV-Rev (Addgene, #12253) and envelope protein VSV-G (Addgene, #8454) using JetPrime (PolyPlus, 114-07). HEK293 media were changed 24 h after transfection and replaced with 35 ml Ultraculture media (Lonza BE12-725F), 350 µl sodium pyruvate (11 mg/ml stock, Thermo Fisher, 11360070), 350 µl sodium butyrate (0.5 M stock, Sigma, B5887), and 350 µl antibiotic/antimycotic (Thermo, 15-240-062) (http://syntheticneurobiology.org/protocols/protocoldetail/31/12). After an additional 48 h, media were collected, concentrated with an ultracentrifuge, and then resuspended in 50–100 µl of sterile PBS.

To confirm that transcribed STICR barcodes can be accurately recovered using scRNA-seq, we performed a ‘barnyard experiment’ in which we infected separate cultures of human cortical cells (GW18 sample) and mouse 3T3 cells (ATCC) with different STICR libraries. These libraries could be distinguished from each other by a constant sequence unique to each library (‘viral index’). After 3 days, we dissociated cultures with papain and FACS-isolated eGFP^+^ cells. eGFP^+^ cells from both species were then mixed together and loaded into a 10X Genomics Chromium Single Cell ’3 prime kit (10x Genomics, PN-100007). Following sequencing, transcript libraries were aligned with CellRanger (version 3.0.2) to a hybrid mouse/human genome and droplets were determined to be either a mouse cell, human cell or multiplet. STICR barcodes were recovered (see below) and the recovered viral index sequence was used to match recovered barcode to the barcode initially used to infect each experiment. Finally, we quantified recovered viral indices for mouse, human and multiplet droplets.

To measure STICR plasmid library barcode diversity, we first digested 1 µg of each library with XhoI and then ligated a PCR adapter containing a unique molecular identifier (UMI) to this site. Ligation products were amplified by PCR using Q5 Hot Start High Fidelity 2x Master Mix (NEB, #M0494) using primers targeting the STICR sequencing primer site and the adapter sequence using the following program: (1) 98 °C for 30 s, (2) 98 °C for 10 s, (3) 62 °C for 20 s, (4) 72 °C for 10 s, (5) repeat steps 2–4 15 times, (6) 72 °C for 2 min, and (7) 4 °C hold. Following PCR amplification, a 0.8–0.6× dual-sided size selection was performed using Ampure XP beads (Beckman Coulter, #A63881). The resulting libraries were sequenced to the depth of approximately 30 million reads. STICR barcode sequences were extracted using custom scripts that removed PCR duplicate reads using the UMI (see below in ‘ScRNA-seq analysis and STICR barcode analysis’ for a general description). Since it is prohibitively expensive to sequence the library to saturation, we extrapolated the total number of unique STICR barcodes using the Preseq^48^ command lc_extrap and default settings. Together with the measured relative barcode abundances, we used the extrapolated STICR barcode library size to model barcode collisions using the R (v4.0.1) programming language. Using base R functions, we simulated the labelling of a starting population of cells with a range of sizes from 10^1^ to 10^6^ and repeated each simulation 20,000 times. We then quantified the mean number of unique barcodes chosen for each starting cell population size. The difference between the starting cell population size and the number of unique barcodes present represented the number of collisions that had happened at that population size.

### TrackerSeq library preparation and validation

TrackerSeq is a *piggyBac* transposon-based^34^ library, developed to be compatible with the 10x single-cell transcriptomic platform. It records the in vivo lineage history of single cells through the integration of multiple oligonucleotide sequences into the mouse genome. Each of these individual lineage barcodes is a 37-bp long synthetic nucleotide that consists of short random nucleotides bridged by fixed nucleotides. This design results in a library with a theoretical complexity of approximately 4.3 million lineage barcodes (16^8^) with each barcode differing from another by at least 5 bp.

To construct the library, the *piggyBac* donor plasmid (Addgene #40973) was altered to include a number of modifications. A Read2 partial primer sequence was cloned into the 3′ UTR of the eGFP to enable retrieval by the 10x platform. The sucrose gene was cloned into the vector, so that empty plasmids that fail to incorporate a lineage barcode during the cloning process are removed. Following digestion with BstXI to remove the sucrose gene, the plasmid was run on a gel and column purified. The lineage barcode oligo mix was cloned downstream of the Read2 partial primer sequence in the purified donor plasmid via multiple Gibson Assembly reactions, as previously described^49^. Gibson assembly reactions (NEB, #E2611S) were then pooled and desalted with 0.025 μm MCE membrane (Millipore, #VSWP02500) for 40 min, and finally concentrated using a SpeedVac. 3 μl of the purified assembly is incubated with 50 μl of NEB10-β-competent *Escherichia coli* cells (NEB, #C3019H) for 30 min at 4 °C, then electroporated at 2.0 kV, 200 Ω, 25 µF (Bio-Rad, Gene Pulser Xcell Electroporation Systems). Electroporated *E. coli* were incubated for 90 min shaking at 37 °C and then plated into pre-warmed sucrose/ampicillin plates. The colonies were scraped off the plates after 8 h, and the plasmids were grown in LB medium with ampicillin up to OD = 0.5. The plasmid library was purified using column purification kit (Zymo Pure II Plasmid Maxiprep kit, #D4202). We first assessed the integrity of the TrackerSeq barcode libraries by sequencing the library to a depth of approximately 42 million reads to test whether any barcode was over-represented. Around 3.6 million valid lineage barcodes that had a quality score of 30 or higher were extracted from the R2 FASTQ files using Bartender^50^. One thousand barcodes were randomly sampled from the extracted lineage barcodes to assess hamming distance. To group similar extracted barcodes into putative barcodes, Bartender assigns a UMI to each barcode read to handle PCR jackpotting errors, and clusters them. The cluster distance was set to 3 so that extracted barcodes within 3 bp of each other have a chance of being clustered together. A total of 2 × 10^5^ clusters of barcodes were identified, suggesting that the barcode library has a diversity that is at least in the 10^5^ range.

### Mice and in utero surgeries

All mouse colonies were maintained in accordance with protocols approved by the Bavarian government at the Max Planck Institute of Neurobiology or the IACUC at the NYU Grossman School of Medicine. Swiss Webster and C57BL/6 wild-type females were used, and embryos were staged in days post-coitus, with E0.5 defined as 12:00 of the day a vaginal plug was detected after overnight mating. Timed pregnant mice were anaesthetized with isoflurane (5% induction, 2.5% during the surgery) and treated with the analgesic Metamizol (WDT). In utero surgery and injection of the STICR lentiviral library in the lateral ventricles of the embryonic mouse forebrains at E10.5–E14.5 were performed as previously described^51^. A microsyringe pump (Nanoject III Programmable Nanoliter Injector (100/240V) (#DRUM3-000-207)) was used to inject approximately 0.5 µl of the STICR library per embryo. For embryos injected at E10.5, ultrasound backscatter microscope (UBM) was used to allow for image-guided injections. For in utero electroporation of the TrackerSeq library, E12.5 embryos were injected unilaterally with 700 nl of DNA plasmid solution made of 0.5 µg/µl pEF1a-pBase (*piggyBac*-transposase; a gift from R. Platt) and the TrackerSeq library 0.5 µg/µl, diluted in endo-free TE buffer and 0.002% Fast Green FCF (Sigma), into the lateral ventricle via a microsyringe pump. Embryos were then electroporated by holding each head between platinum-plated tweezer electrodes (5 mm in diameter, BTX, #45-0489) across the uterine wall, while 5 electric pulses (35 V, 50 ms at 1 Hz) were delivered with a square-wave electroporator (BTX, ECM 830)^52^. Pregnant dams were kept in single cages and pups were kept with their mothers, in the institutional animal facility under standard 12:12-h light–dark cycles, at a room temperature of 72° F ± 2° F and a humidity of 30–70%.

### Sample collection

Virally injected brains were collected from mouse pups between ages postnatal day 5 and day 15 (P5–P15) (Supplementary Data 1). Brains were dissected in ice-cold pre-bubbled artificial cerebrospinal fluid (aCSF), and sectioned into 400-µm coronal sections on a Leica VT1200S Vibratome. Coronal brain sections were then dissected such that the forebrain was collected, thus excluding the thalamus, hypothalamus, brainstem and cerebellum. Alternatively, OBs, amygdalae and striata were manually dissected out from sliced brains, and processed separately. Collected tissue was then dissociated with the Miltenyi BioTech Neural Tissue Dissociation Kit (P) (#130-092-628) on the gentleMACS Dissociator according to the protocol of the manufacturer. To isolate and collect virally infected cells, flow cytometry was done using a SY3200 Cell Sorter (software WinList 3D version 8.0.) or BD FACSAria III Cell Sorter (BD FACSDiva Software, version 8.0.2) with a 100-µm nozzle. The cell suspensions were first gated on forward scatter, then within this population based on eGFP expression. eGFP-expressing cells were collected in bulk for downstream processing on the 10x Genomics Chromium platform.

Ganglionic eminences were collected from mouse embryos at E13.5 and E15.5 (Supplementary Data 1) according to the following protocol: embryos were removed from the uterus of wild-type Swiss Webster females, and stored in ice-cold L-15 medium. Brains were removed from the embryonic skulls, and the MGE, CGE and LGE were dissected out. MGEs, CGEs and LGEs were then each pooled together from multiple embryos, so that each eminence type was processed independently, and dissociated with the Miltenyi BioTech Neural Tissue Dissociation Kit (P) (#130-092-628) on the gentleMACS Dissociator according to the protocol of the manufacturer.

For embryonic lineage tracing, we collected electroporated brains from mouse embryos at E16.5 (Supplementary Data 1) in Leibowitz medium with 5% FBS. Papain dissociation system was carried out according to the recommended protocol (Wortington, #LK003150), and to isolate positive cells, flow cytometry was done using a BD FACSAria III Cell Sorter (BD FACSDiva Software, version 8.0.2) with a 100-µm nozzle. For all FACS experiments, non-eGFP-expressing brain tissue was used as a negative control for excluding background fluorescence.

### Immunohistochemistry

E14.5 and P10 mice were perfused with 4% PFA and post-fixed overnight in 4% PFA at 4 °C. Coronal sections (60 µm) were performed using vibrating microtome. Immunofluorescent staining was performed as follows: sections were incubated for 1 h at room temperature in blocking solution (5% BSA and 0.3% Triton-X100 in PBS), then overnight at 4 °C with primary antibodies. Sections were rinsed three times in PBS 1X and incubated for 1 h at room temperature with the corresponding secondary antibody (1:500, Life Technologies). Three washes with PBS 1X were performed, the second wash using Hoechst staining solution (1:10,000 in PBS 1X, Invitrogen) to label nuclei, before dry mounting on slides with Fluoromount-G (Invitrogen). For imaging, the primary somatosensory area was used as region of study. Images were acquired on a Leica Sp8 confocal laser scanning microscope.

The primary antibodies used included: rabbit anti-CUX1 1:500 (Santa Cruz, #SC13024), rabbit anti-GABA 1:2,000 (Merck, #A2052), rabbit anti-GFP 1:1,000 (Invitrogen, #A11122), rabbit anti-Iba1 1:500 (Wako, #019-19741), rabbit anti-OLIG2 1:500 (Merck, #AB9610), rabbit anti-S100β 1:500 (Merck, #S2644), and rat anti-CTIP2 [25B6] 1:500 (Abcam, #AB18465).

The secondary antibodies used were: 647 Alexa Fluor plus goat anti-rabbit (Invitrogen, #A32733), 555 Alexa Fluor goat anti-rat (Invitrogen, #A21434), and 555 Alexa Fluor goat anti-rabbit (Invitrogen, #A21428).

### Preparation of RNA-seq, STICR and TrackerSeq libraries

For experiments utilizing the 10x Genomics platform, the following reagents were used: Chromium Single Cell 3′ Library & Gel Bead Kit v2 (PN-120237), Chromium Single Cell 3′ Chip Kit v2 (PN-120236) and Chromium i7 Multiplex Kit (PN-120262) were used according to the manufacturer’s instructions in the Chromium Single Cell 3′ Reagents Kits V2 User Guide; Chromium Single Cell 3′ Library & Gel Bead Kit v3 (PN-1000075), Chromium Single Cell 3′ Chip Kit V3 (PN-1000073) and Chromium i7 Multiplex Kit (PN-120262) were used according to the manufacturer’s instructions in the Chromium Single Cell 3′ Reagents Kits V3 User Guide; Chromium Single Cell 3′ Library & Gel Bead Kit v3.1 (PN-1000268), Chromium Single Cell 3′ Chip Kit V3.1 (PN-1000127) and Dual Index Kit TT Set A (PN-1000215) were used according to the manufacturer’s instructions in the Chromium Single Cell 3′ Reagents Kits V3.1 User Guide (Dual Index).

The lineage barcode library retrieved from RNA was amplified with a standard NEB protocol for Q5 Hot Start High-Fidelity 2X Master Mix (#M094S) in a 50-µl reaction, using 10 µl of cDNA as template. Specifically, each PCR contained: 25 µl Q5 High-fidelity 2X Master Mix, 2.5 µl 10 µM P7_indexed reverse primer, 2.5 µl 10 µM i5_indexed forward rimer, 10 µl molecular grade H_2_0, 10 µl cDNA (for primer sequences and indices, see Supplementary Data 1). The PCR protocol for amplifying STICR lineage libraries was: (1) 98 °C for 30 s, (2) 98 °C for 10 s, (3) 62 °C for 20 s, (4) 72 °C for 10 s, (5) repeat steps 2–4 11–18 times, (6) 72 °C for 2 min, and (7) 4 °C hold. The PCR protocol for amplifying TrackerSeq lineage libraries was: (1) 98 °C for 30 s, (2) 98 °C for 10 s, (3) 63 °C for 20 s, (4) 72 °C for 10 s, (5) repeat steps 2–4 11–18 times, (6) 72 °C for 2 min, and (7) 4 °C hold. Libraries were purified with a dual-sided SPRI selection using Beckman Coulter Agencourt RNAClean XP (A63987), and quantified with an Agilent BioAnalyzer. Some STICR libraries (DI_T_199, DI_T_203, DI_T_211, DI_T_222, DI_T_233, DI_T_238, DI_T_239, DI_T_240, DI_T_241, DI_T_242, DI_T_287, DI_T_289, DI_T_304 and DI_T_305) were constructed and sequenced twice to achieve higher resolution.

### Sequencing and read mapping

Transcriptome and barcode libraries were sequenced either on an Illumina NextSeq 500 at the Next Generation Sequencing Facility of the Max Planck Institute of Biochemistry, at the Genomics Core Facility at the Helmholtz Center in Munich, or on a NovaSeq at the Broad Institute. For a detailed report on each dataset, see Supplementary Data 1. Sequencing reads in FASTQ files were aligned to a reference transcriptome (mm10-2.1.0) and collapsed into UMI counts using the 10x Genomics Cell Ranger software (version 3.0.2 or 5.0.1).

### Processing of STICR barcode reads

STICR barcode analysis was performed using custom scripts. First, BBMap (BBMap—Bushnell B.; sourceforge.net/projects/bbmap/) was used to remove low-quality reads and then extract reads containing STICR barcode sequences. Then, BBMap was used to extract individual STICR barcode fragments, which were then aligned to our pre-defined fragment reference sets using Bowtie (v5.2.1)^53^, allowing for up to two mismatches per fragment. Aligned STICR barcodes were compiled into a file containing their corresponding 10X cell barcode and 10X UMI sequences using Awk. Finally, UMI-tools (v.0.5.1)^54^ was used to remove duplicate STICR barcode or cell barcode reads by UMI, allowing for 1 bp mismatch in the UMI. STICR barcodes–CBC pairings with at least five distinct UMIs were retained for clonal analysis. Among cells with multiple STICR barcodes passing these criteria, we attempted to find a ‘dominant’ STICR barcode that we defined as containing greater than or equal to five times the number of UMI counts than the next most abundance STICR barcode. Dominant STICR barcodes meeting these criteria were considered to be the clonal barcode of their respective cells and retained for further analysis. Within metadata files, these CBC–STICR barcode pairings are referred to as tier 2, whereas CBC–STICR barcode pairs with only a single STICR barcode meeting threshold criteria are referred to as tier 1. Only CBCs associated with a single STICR barcode tier 1) were used in this study, with the exception of the OB–amygdala–striatum experiment in Extended Data Fig. 8, where tier 2 was used. Finally, we applied an additional UMI threshold to STICR barcodes, requiring that all STICR barcode–CBC pairings used for clonal analysis have at least nine distinct UMIs. Three STICR barcodes (Index1_Bit1_F_083-Bit2_F_060-Bit3_F_055,_Bit1_F_057-Bit2_F_103-Bit3_F_244 and IndexE_Bit1_F_246-Bit2_F_178-Bit3_F_497) were removed from downstream analyses, because they were present in more than one dataset. Two of the total 21 STICR datasets (CA199 and CA233) contained only single-cell clones. These datasets were retained to support single-cell cluster analysis and clone size quantification.

### Processing of TrackerSeq barcode reads

Reads in the R2 FASTQ files were pre-processed so that the sequences to the left and right of the lineage barcodes (BC) were trimmed. Lineage barcodes shorter than 37 bp were discarded. Cell barcodes (Cell) were extracted from the corresponding Seurat object of the dataset to generate a cell barcode whitelist. The extracted cell barcodes and UMIs were added to the read names of the lineage barcode FASTQ files. The resulting FASTQ files were processed to output a sparse matrix in csv format, where rows were cells identified by individual cell barcodes and columns were lineage barcodes. Only Cell–UMI–BC triples supported by at least 10 reads and Cell–BC pairs with at least 6 UMI were considered for further analyses. CloneIDs were assigned to cell barcodes by clustering the matrix using Jaccard similarity and average linkage as demonstrated by Wagner and colleagues^24^. The resulting dendrogram was cut at a height of 0.999 to obtain the clonal groupings. The clonal groupings showed that there were 4,282 barcodes distributed over 2,370 cells in the total dataset, where 56.0% of them were marked by 2 or more barcode integrations, and 8.4% of them were marked by 5 or more integrations in the total dataset. Among the inhibitory neurons featured in Fig. 3, these numbers were 85.7% and 9.5%, respectively.

### Cell filtering, data normalization, batch correction and clustering of STICR datasets

The Seurat workflow (version 3.1.4) was used for cell filtering, data normalization and cluster identification in scRNA-seq datasets. Data were read into R (version 3.6.0) as a count matrix. Each dataset was filtered with cut-offs for: maximum and minimum gene expression, maximum nCount_RNA, and the percentage of total reads that aligned to the mitochondrial genome (for applied cut-offs, see Supplementary Data 1). Filtered data were then used for standard processing with Seurat. Unless otherwise indicated, gene expression values for each cell were divided by the total number of transcripts and multiplied by 10,000. These values were then log transformed using log1p via the NormalizeData() function. Genes were scaled and centred using the ScaleData() function. We used Harmony (v1.0)^28^ within the Seurat workflow with default parameters (theta = 2, lambda = 1, sigma = 0.1) to integrate different STICR datasets. We used the first 35 Harmony embeddings for UMAP (https://github.com/lmcinnes/umap) visualizations and clustering analysis.

To partition cells into clusters, we constructed a shared-nearest neighbour graph based on Harmony embeddings via the FindNeighbors() function to use as input to the SLM algorithm, implemented through the FindClusters() function in Seurat (dimensions = 35, res = 1) . Cluster-specific marker genes were identified by comparing cells of each cluster to cells from all other clusters. Genes were considered differentially expressed based on fold change, minimum expression and adjusted *P* value cut-offs (Supplementary Data 3). The Wilcoxon rank sum test was implemented via the Seurat function FindAllMarkers().

Clusters were manually annotated based on marker gene expression, spatial transcriptome mapping, as well as publicly available databases, primarily DropViz (dropviz.org)^55^ and Mouse Brain Atlas (http://mousebrain.org/genesearch.html)^56^. Of the 41 unsupervised STICR clusters, 9 clusters were reclustered to gain a higher level of detail (for example, cluster 7 was split into clusters 7a and 7b; Fig. 3a). More specifically, clusters were isolated using subset() and clustered again using FindClusters(). Sub-clusters that could not be assigned to a cell type were assigned ‘unknown’ and excluded from the lineage analysis (0.76% of the total cells). Cluster 22, which contained sub-clusters of different classes, was labelled ‘mixed’. All analyses were carried out based on refined clusters, except for panels illustrating the spatial analysis.

### Lineage analysis of cell classes in STICR

To quantify clonal relationships between cell classes, Seurat clusters were merged into cell classes (Fig. 1d) based on the co-expression of multiple marker genes (neurons (*Tubb3* and *Mef2c*); neuronal precursors (*Gad1* and *Neurod2*); mitotic cells (*Ube2c* and *Top2a*); astrocytes (*Aldh1l1* and *Gfap*); oligodendrocytes (*Olig1* and *Plp1*); OPCs (*Pdgfra* and *C1ql1*); vascular cells (*Rgs5*); epithelial cells (*Ttr*); ependymal cells (*Tmem212*); and macrophages (*Ccl4* and *C1qa*)) (Extended Data Fig. 2a, Supplementary Data 2). Clones were categorized as containing sister cells that were glia only (astrocyte, OPC and oligodendrocyte classes), neuron only, or glia and neuron mix (neuron, astrocyte, OPC and oligodendrocyte classes), and the number of clones in each of these three categories was quantified relative to the total number of clones at each developmental stage.

### Lineage coupling z-scores and correlations forebrain-wide STICR datasets

The numbers of shared clones, as well as lineage coupling z-scores and correlations were calculated for each pair of cell states based on the methods outlined by Wagner and colleagues^24^ as follows:

1. Definitions:
  For a given clone *c*_1_ with *n* cells, and a given cell-state pair {*s*_1_, *s*_2_}:
  1.1. Let *k* be the number of cells of clone *c*_1_ that were assigned to either of the cell states of the pair, that is, *s*_1_ or *s*_2_.
  1.2. Clone *c*_1_ is defined to be ‘shared’ between states *s*_1_ and *s*_2_ if *k* ≥ 2, and there was at least one cell of clone *c*_1_ assigned to each state.
  1.3. Let *p* be the fraction of clone *c*_1_ that *k* represents, that is, *p* = *k* /*n*.
  1.4. A metric for the cell-state pair {*s*_1_, *s*_2_} is defined as the sum, over all ‘shared’ clones, of the *p* of each clone.
2. The metric defined in (1.4) was computed for each pair of cell states, according to the observed data.
3. A distribution of values of the metric was computed for each pair of cell states in the following way:
  3.1. For *N* = 10,000 iterations, the following simulation was done:
    3.1.1. Maintaining the observed distribution of the number of cells per cell state, the state assignments of the individual cells were randomly shuffled.
    3.1.2. The metric defined in (1.4) was computed for each pair of cell states, according to the data resulting from this simulation.
4. For each pair of cell states, its lineage coupling z-score is defined as the z-score of its observed metric computed in (2), with respect to the distribution computed in (3).
  Positive *z*-scores indicate pairs of cell states that shared more lineage barcodes than expected by chance, whereas a negative score indicates that a state pair was significantly less coupled than expected by chance.
5. For each pair of cell states, its lineage coupling correlation is defined as the correlation between all the lineage coupling z-scores of each individual cell state of the pair.

### Dendrograms and UpSet plots

Dendrograms representing transcriptomic relationships were generated with the BuildClusterTree() function in Seurat, which constructs a phylogenetic tree relating the ‘average’ cell from each identity cluster. The tree is estimated on the basis of a distance matrix constructed in the gene expression space. Dendrograms representing lineage relationships were generated using the hclust() and dist() functions on lineage coupling correlations, with an average linkage clustering and Euclidean distance metric. The interrelation between cell types can only be coarsely represented in hierarchical dendrograms. Dendrograms represent overall transcriptomic similarities and dissimilarities, but they fail to capture less obvious similarities between otherwise distinct cell types. Similarly, dendrograms may represent the general nexus of clonal relationships but overlook infrequent relationships.

UpSet plots were created in R using the UpSetR library^57^. For set size, we used the number of cells per cluster.

### Correlation-based distance measure for amygdala, OB and striatum datasets

Amygdala, OB and striatum datasets were pre-processed as mentioned above. Cell types were manually annotated and neuronal types were divided via subset(). The distance between the log-normalized average cluster gene expression was calculated using the Spearman correlation-based distance measure in the get_dist() function and visualised using fviz_dist() from the R package factoextra v1.0.7.

### Cell filtering, data normalization batch correction and clustering of embryonic datasets

The Seurat pipeline (version 3.1.4) was used for cluster identification in scRNA-seq datasets. Embryonic transcriptome datasets (MUC28072, CA303, CA300, CA302, CA299, CA301 and CA298) were read into R (version 3.6.0) as a count matrix. Each dataset was filtered with cut-offs for: maximum or minimum gene expression, maximum nCount_RNA and the percentage of total reads that aligned to the mitochondrial genome (for applied cut-offs, see Supplementary Data 1). In addition, embryonic datasets were filtered with DoubletFinder version 2.0.3 (ref.^58^).

We used regularized negative binomial regression^59^ to normalize UMI count data for all embryonic datasets. Cells with UMI counts for Neurod2 > 2 and Neurod6 > 2, which are markers of excitatory neurons, were removed. The TrackerSeq dataset was clustered using Seurat standard procedures and clusters expressing marker genes for excitatory neurons were removed. We created an ‘integrated’ data assay including all embryonic datasets for downstream analysis as described by Stuart and colleagues^60^. Clusters of cells were identified by a shared nearest neighbour modularity optimization-based clustering algorithm. Uniform manifold approximation and projection (UMAP) dimensional reduction (https://github.com/lmcinnes/umap) was applied to the integrated data assay for visualization.

### Trajectory analysis of embryonic datasets

Trajectory inference and pseudotime calculations were done with Monocle3 (ref.^35^). RNA velocity was estimated using the R library velocyto.R^36^. 10x output files were preprocessed with velocyto, version 0.17.17 (https://velocyto.org) using the command velocyto run10x. The velocyto.R-package ‘velocyto.R’ version 0.6 was used for RNA velocity estimation in R.

### Mapping embryonic cells to postnatal clusters

To map cells from embryonic trajectories to postnatal cell types, we first selected the five embryonic Seurat clusters from the ‘integrated’ data assay that were located at the tip of the Monocle trajectories, as well as Seurat clusters from the postnatal STICR dataset that were identified and annotated as subpallial GABAergic neuron types. We focused on 1,855 genes that were identified as variable features at both developmental stages using the Seurat FindVariableFeatures() function. For these genes, we averaged the log-normalized expression in the postnatal clusters to create postnatal cell-type model vectors. We then calculated Pearson correlations between all individual cells of the embryonic clusters and the model vectors as described in Mayer et al^1^. We assigned each cell to the postnatal cluster with the highest correlation, but also calculated empirical *P* values to determine the significance of the assignment by permuting the single-cell data for a random background. We left the model vectors unchanged, but permuted the single-cell expression data 100 times. For each permutation and each cell, we kept track of the largest Pearson correlation to the model vectors, and calculated a *P* value for the cluster assignment by counting what fraction of correlation scores was larger than the one used for the cluster assignment. In a final step, we turned all *P* values into false discovery rates (FDRs) and mapped only cells with an FDR < 0.1 to the postnatal clusters.

### Spatial gene expression in STICR

To infer the spatial location of the clusters, the STICR datasets were integrated with the Visium Spatial Transcriptomic datasets for sagittal and coronal sections of the mouse brain provided by 10x genomics (https://support.10xgenomics.com/spatial-gene-expression/datasets). We applied an ‘anchor’-based integration workflow in Seurat v3, which enables the probabilistic transfer of annotations from a reference to a query set. The spatial reference dataset and the lineage dataset were normalized using the SCTransform() function, which builds regularized negative binomial models of gene expression, and performed dimensionality reduction using the RunPCA() function and then performed label transfer using the functions FindTransferAnchors() and TransferData(). This procedure outputs, for each spatial spot, a probabilistic classification for each of the scRNA-seq-derived cell states. We added these predictions as a new assay in the Seurat object for visualization using the function SpatialFeaturePlot().

### Scatter plots

The top 100 marker genes were calculated using the Seurat function FindMarkers () for a selection of GABAergic clusters in the postnatal STICR dataset and the merged embryonic dataset, respectively (postnatal: ‘2 Inhibitory neuron OB *Meis2*’, ‘6 Inhibitory neuron OB *Synpr*’, ‘7a D2 SPNs’, ‘7b D1 SPNs’, ‘8 Inhibitory ITC amygdala’, ‘34 Inhibitory PN ventral striatum/central extended amygdala (EAC)’; ‘13a MGE IN *Snhg11*’, ‘19a CGE VIP IN’, ‘19b CGE neurogliaform IN’, ‘13a MGE IN *Snhg11*’; embryonic: ‘i_*Six3*/*Gucy1a3*’, ‘i_*Ebf1*/*Isl1*’, ‘i_*Phlda1*/*Isl1*’, ‘i_*Nr2f2*’, ‘i_*Nxph1*’). On the basis of the correlation-based mapping of embryonic cells to postnatal clusters (see previous paragraph), we selected pairs of embryonic and adult clusters for the scatter plot. We plotted the SCT normalized average cluster gene expression of the top 100 marker genes from each stage.

### Reporting summary

Further information on research design is available in the Nature Research Reporting Summary linked to this paper.

## Online content

Any methods, additional references, Nature Research reporting summaries, source data, extended data, supplementary information, acknowledgements, peer review information; details of author contributions and competing interests; and statements of data and code availability are available at 10.1038/s41586-021-04237-0.

## Supplementary information

Reporting Summary

Supplementary Data 1 Descriptive report of collected datasets.

Supplementary Data 2 Top 20 differentially expressed genes for cell classes.

Supplementary Data 3 Top 20 differentially expressed genes for cell type clusters.

Supplementary Data 4 Top 20 differentially expressed genes for embryonic clusters.

## Data Availability

The sequencing datasets generated for the current study are available in the Gene Expression Omnibus (GEO) under the accession number GSE188528. Publicly available gene expression data used for cluster annotation can be accessed as follows: DropViz (dropviz.org) and Mouse Brain Atlas (http://mousebrain.org/genesearch.html). Visium Spatial Transcriptomic Datasets for sagittal and coronal sections of the mouse brain provided by 10x genomics (https://support.10xgenomics.com/spatial-gene-expression/datasets).

## Code availability

The analyses described here are available on GitHub: https://github.com/mayer-lab/Bandler-et-al_lineage.
