# Supplementary material for: Single-cell delineation of lineage and genetic identity in the mouse brain
Source: Nature. 2021 Dec 15;601(7893):404–9. doi: 10.1038/s41586-021-04237-0 (PMC8770128; doi:10.1038/s41586-021-04237-0)
Supplement: Supplementary file 1 — Reporting Summary [file 41586_2021_4237_MOESM1_ESM.pdf]

Corresponding author(s): Christian Mayer
Last updated by author(s): Nov 3, 2021

Reporting Summary

Nature Portfolio wishes to improve the reproducibility of the work that we publish. This form provides structure for consistency and transparency in reporting. For further information on Nature Portfolio policies, see our Editorial Policies and the Editorial Policy Checklist.

Statistics

For all statistical analyses, confirm that the following items are present in the figure legend, table legend, main text, or Methods section.

n/a	Confirmed	
☐	☒	The exact sample size (*n*) for each experimental group/condition, given as a discrete number and unit of measurement
☐	☒	A statement on whether measurements were taken from distinct samples or whether the same sample was measured repeatedly
☐	☒	The statistical test(s) used AND whether they are one- or two-sided<br>*Only common tests should be described solely by name; describe more complex techniques in the Methods section.*
☐	☒	A description of all covariates tested
☐	☒	A description of any assumptions or corrections, such as tests of normality and adjustment for multiple comparisons
☐	☒	A full description of the statistical parameters including central tendency (e.g. means) or other basic estimates (e.g. regression coefficient) AND variation (e.g. standard deviation) or associated estimates of uncertainty (e.g. confidence intervals)
☐	☒	For null hypothesis testing, the test statistic (e.g. *F*, *t*, *r*) with confidence intervals, effect sizes, degrees of freedom and *P* value noted<br>*Give P values as exact values whenever suitable.*
☒	☐	For Bayesian analysis, information on the choice of priors and Markov chain Monte Carlo settings
☐	☒	For hierarchical and complex designs, identification of the appropriate level for tests and full reporting of outcomes
☐	☒	Estimates of effect sizes (e.g. Cohen's *d*, Pearson's *r*), indicating how they were calculated

Our web collection on statistics for biologists contains articles on many of the points above.

Software and code

Policy information about availability of computer code

Data collection	Flow cytometry was done using a SY3200 Cell Sorter (software WinList 3D version 8.0.) or BD FACSAria III Cell Sorter (BD FACSDiva Software, version 8.0.2).
Data analysis	The following software/packages were utilized in the manuscript: 10x Genomics Cell Ranger software (version 3.0.2 and 5.0.1), Seurat (version 3.1.4), R (version 3.6.0 and version 4.0.1), DoubletFinder (version 2.0.3), Harmony (version 1.0), UMAP (https://github.com/lmcinnes/umap, RNA velocity (R library velocyto.R, version 0.17.17, https://velocyto.org), Monocle (version 3), Bartender (version 1.1, https://github.com/LaoZZZZZ/bartender-1.1), BBMap (version 38.67, BBMap – Bushnell B. – sourceforge.net/projects/bbmap/), Bowtie (v5.2.1), UMI-tools (v.0.5.1), LARRY (https://github.com/AllonKleinLab/LARRY, modified from last commit on 31 Oct 2018). All in-house scripts for analysis are available at https://github.com/mayer-lab/Bandler-et-al_lineage.

For manuscripts utilizing custom algorithms or software that are central to the research but not yet described in published literature, software must be made available to editors and reviewers. We strongly encourage code deposition in a community repository (e.g. GitHub). See the Nature Portfolio guidelines for submitting code & software for further information.

Data

Policy information about availability of data

All manuscripts must include a data availability statement. This statement should provide the following information, where applicable:

- Accession codes, unique identifiers, or web links for publicly available datasets
- A description of any restrictions on data availability
- For clinical datasets or third party data, please ensure that the statement adheres to our policy

The sequencing datasets generated for the current study are available in the Gene Expression Omnibus (GEO) at GEO accession number. Publicly available gene expression data used for cluster annotation can be accessed as follows: DropViz (dropviz.org) and Mouse Brain Atlas (http://mousebrain.org/genesearch.html). Visium Spatial Transcriptomic Datasets for sagittal and coronal sections of the mouse brain provided by 10x genomics (https://support.10xgenomics.com/spatial-gene-expression/datasets).

Field-specific reporting

Please select the one below that is the best fit for your research. If you are not sure, read the appropriate sections before making your selection.

☒ Life sciences ☐ Behavioural & social sciences ☐ Ecological, evolutionary & environmental sciences

For a reference copy of the document with all sections, see nature.com/documents/nr-reporting-summary-flat.pdf

Life sciences study design

All studies must disclose on these points even when the disclosure is negative.

Sample size	No statistical methods were used to predetermine sample sizes. After filtering out low-quality cells, the following number of cells was analysed: Postnatal (STICR) analyses: 65700 cells; Postnatal (STIRC_AOS) analyses: 12519 cells; Embryonic analyses: 29380 cells.
Data exclusions	During sub-cluster assignment, cells that could not be assigned to a cell type based on marker gene expression were assigned "unknown" and excluded from the lineage analysis (0.76% of the total cells). For analysis of the embryonic datasets, which focused on inhibitory lineages, excitatory neurons were removed. For more details, see methods sections.
Replication	Postnatal (STICR) analyses: 18 samples including 25 brains; Postnatal (STIRC_AOS) analyses: 3 samples including 10 brains; Embryonic analyses: 7 samples including 14 brains.
Randomization	Not relevant to this study; no need to prevent selection bias or bias in treatment assignments.
Blinding	Not relevant to this study; no need to prevent selection bias or bias in treatment assignments.

Reporting for specific materials, systems and methods

We require information from authors about some types of materials, experimental systems and methods used in many studies. Here, indicate whether each material, system or method listed is relevant to your study. If you are not sure if a list item applies to your research, read the appropriate section before selecting a response.

Materials & experimental systems

n/a	Involved in the study	
☐	☒	Antibodies
☐	☒	Eukaryotic cell lines
☒	☐	Palaeontology and archaeology
☐	☒	Animals and other organisms
☒	☐	Human research participants
☒	☐	Clinical data
☒	☐	Dual use research of concern

Methods

n/a	Involved in the study	
☒	☐	ChIP-seq
☐	☒	Flow cytometry
☒	☐	MRI-based neuroimaging

Antibodies

Antibodies used	Primary antibodies used are: rabbit anti-CUXl 1-500 (Santa Cruz, #SC13024), rabbit anti-GABA 1:2000 (Merck, #A2052), rabbit anti-lbal 1:500 (Wako, #019-19741), rabbit anti-OLIG2 1:500(Merck, #AB9610), rabbit anti-SlOOb 1:500 (Merck, #52644), rat anti-CTIP2 [25B6] 1:500 (Abcam,#AB18465). Secondary antibodies used: 647 Alexa Fluor Plus goat anti-rabbit (Invitrogen, #A32733), 555 Alexa Fluor goat anti-rat (invitrogen, #A21434), 555 Alexa Fluor goat anti-rabbit (Invitrogen, #A21428).

Validation	Below please find description of validation for all primary antibodies used in this study:<br>Rabbit anti-CUXl (SC, 5(13024) is a rabbit polyclonal lgG, whose epitope corresponds to amino-acids 1111-1332 mapping at the (terminus of mouse Cuxl protein. Previously used to label excitatory neurons of upper cortical layers (Mattugini et al., Neuron, 2019, 103: 1086-95).<br>Rabbit anti-GABA (Merck, A2052) is produced in rabbit affinity isolated antibody, previously used to label inhibitory GABAergic neurons (Teissier et al., J. Neurosci., 2010, 30(31): 10563-74).<br>Rabbit anti-lbal (Wako #019-19741) is a rabbit polyclonal antibody largely used as standard marker for labelling microglia (Mattugini et al., Neuron, 2019, 103: 1086-95).<br>Rabbit anti-OLIG2 (Merck AB9610) is a purified rabbit polyclonal antibody, previously used to label oligodendrocytes in the brain in IHC (Teissier et al., J. Neurosci., 2010, 30(31): 10563-74).<br>Rabbit anti-Sl00b (Merck, 52644) is a rabbit polyclonal antibody largely used as standard marker for labelling astrocytes (Bengoetxea et al., Front. Cell. Neurosci., 2013, 7:170).<br>Rat anti-CTIP2 (Abeam AB18465) is a rat monoclonal antibody, previously used to label excitatory neurons of lower cortical layers (Mattugini et al., Neuron, 2019, 103: 1086-95).

Eukaryotic cell lines

Policy information about cell lines

Cell line source(s)	Lenti-X HEK293T (Takara Bio)
Authentication	Cell lines were not authenticated.
Mycoplasma contamination	Cell Lines were not tested for mycoplasma.
Commonly misidentified lines<br>(See ICLAC register)	No commonly misidentified lines used.

Animals and other organisms

Policy information about studies involving animals; ARRIVE guidelines recommended for reporting animal research

Laboratory animals	See Methods section "Mice and In Utero Surgeries." Swiss Webster Wild Type Females and C57BL/6 Wild Type Females (between 6-10 weeks in age) were used for timed pregnant in-utero injections. In utero surgery and injection of STICR library were performed on embryonic mouse forebrains at E10.5, E12.5, E13,5 and E14.5. Virally injected brains were collected from mouse pups between ages postnatal day 5 to 15 (P5-15). For in utero electroporation of the TrackerSeq library, E12.5 embryos were used, and forebrains were collected at E16.5. For collection of ganglionic eminences, embryos were collected at E13.5 and E15.5. Pregnant dams were kept in single cages and pups were kept with their mothers, in the institutional animal facility under standard 12: 12 h light / dark cycles, room temp: 72° +/- 2° F, humidity: 30%-70%.
Wild animals	This study did not involve wild animals.
Field-collected samples	This study did not involve samples collected from the field.
Ethics oversight	All mouse colonies were maintained in accordance with protocols approved by the IACUC at the NYU Grossman School of Medicine and the Bavarian government at the Max Planck Institute of Neurobiology.

Note that full information on the approval of the study protocol must also be provided in the manuscript.

Flow Cytometry

Plots

Confirm that:

☒ The axis labels state the marker and fluorochrome used (e.g. CD4-FITC).

☒ The axis scales are clearly visible. Include numbers along axes only for bottom left plot of group (a 'group' is an analysis of identical markers).

☒ All plots are contour plots with outliers or pseudocolor plots.

☐ A numerical value for number of cells or percentage (with statistics) is provided.

Methodology

Sample preparation	Please see "Sample Collection" section of the methods section.<br>For STICR datsets, brains were dissected in ice-cold pre-bubbled aCSF (Artificial Cerebrospinal Fluid), and sectioned into 400 µm coronal sections on a Leica VT1200S Vibratome. Coronal brain sections were then dissected such that the forebrain was collected. Alternatively, OBs, amygdalae and striata were manually dissected out from sliced brains, and processed separately. Collected tissue was then dissociated with the Miltenyi BioTech Neural Tissue Dissociation Kit (P) (#130-092-628) on the gentleMACS Dissociator according to the protocol of the manufacturer.<br>For embryonic TrackerSeq dataset, we collected E12.5-electroporated brains from 2 mouse embryos at E16.5 in Leibowitz

	medium with 5% FBS. Papain dissociation system was carried out according to the recommended protocol (Wortington, #LK003150).
Instrument	SY3200 Cell Sorter and BD FACSAria III Cell Sorter
Software	WinList 3D version 8.0., BD FACSDiva Software, version 8.0.2
Cell population abundance	Cell population abundance was less than 1% of the total cells sorted.
Gating strategy	The cell suspensions were first gated on forward scatter, then within this population based Dapi to exclude dead cells, and finally on eGFP expression.

☒ Tick this box to confirm that a figure exemplifying the gating strategy is provided in the Supplementary Information.
